# Economic implications of reducing caesarean section rates – Analysis of two health systems

**DOI:** 10.1371/journal.pone.0228309

**Published:** 2020-07-28

**Authors:** Patrick S. Moran, Charles Normand, Patricia Gillen, Francesca Wuytack, Michael Turner, Cecily Begley, Deirdre Daly

**Affiliations:** 1 School of Nursing and Midwifery, Trinity College Dublin, Dublin, Ireland; 2 Centre for Health Policy and Management, Trinity College Dublin, Dublin, Ireland; 3 Cicely Saunders Institute, King’s College London, London, United Kingdom; 4 School of Nursing Research, Institute of Nursing and Health Research, Ulster University, Coleraine, United Kingdom; 5 School Of Medicine, University College Dublin, Dublin, Ireland; La Trobe University, AUSTRALIA

## Abstract

Caesarean section (CS) rates throughout Europe have risen significantly over the last two decades. As well as being an important clinical issue, these changes in mode of birth may have substantial resource implications. Policy initiatives to curb this rise have had to contend with the multiplier effect of women who had a CS for their first birth having a greater likelihood of requiring one during subsequent births, thus making it difficult to decrease CS rates in the short term. Our study examines the long-term resource implications of reducing CS rates among first-time mothers, as well as improving rates of vaginal birth after caesarean section (VBAC), among an annual cohort of women over the course of their most active childbearing years (18 to 44 years) in two public health systems in Europe. We found that the economic benefit of improvements in these two outcomes is considerable, with the net present value of the savings associated with a five-percentage-point change in nulliparous CS rates and VBAC rates being €1.1million and £9.8million per annual cohort of 18-year-olds in Ireland and England/Wales, respectively. Reductions in CS rates among first-time mothers are associated with a greater payoff than comparable increases in VBAC rates. The net present value of achieving CS rates comparable to those currently observed in the best performing Scandinavian countries was €3.5M and £23.0M per annual cohort in Ireland and England/Wales, respectively.

## Introduction

The rate of caesarean section (CS) has been increasing worldwide for over two decades. In Western Europe, average CS rates rose from 19.6% in 2000 to 26.9% in 2015, representing an average increase of 2.1% per year. [1] This increasing trend has been replicated in both Ireland and England/Wales, with overall CS rates having reached 32.7% and 27.8%, respectively, in 2016. [2,3] While CS can be a life-saving intervention when medically indicated, once levels exceed 10% to 15% there is no evidence of a corresponding reduction in maternal or newborn mortality rates. [4] CS is, however, associated with a greater risk of experiencing health problems in later pregnancies such as uterine rupture, ectopic pregnancy, stillbirth, and preterm birth, compared with vaginal birth. [5]

In addition to the risks to individuals, overuse of CS has the potential to cause net harms at a societal level, if the incremental benefits that society foregoes in order to fund the continuous rise in CS rates exceed the incremental benefits that rising CS rates deliver. This opportunity cost of high rates of CS has previously been highlighted as a problem in overloaded or weak health systems, where such activity can divert resources from existing services. [6] Although this phenomenon may be more visible in less well-resourced settings, this is a cost that is incurred in all countries where CS rates exceed medically indicated levels. Even in more affluent countries, having to fund suboptimally high CS rates may curtail policymakers’ ability to introduce or extend other services and divert skilled medical resources from more important uses.

The overall CS rate observed within a given population can be considered a function of CS rates in three distinct subpopulations; 1) nulliparous women, 2) multiparous women with a history of CS, and 3) multiparous women without a history of CS. Efforts to reduce overall CS rates requires consideration of the relative effect of changes in each of these, as well as any ripple effects over time. For instance, decreasing CS rates in first-time mothers will decrease multiparous CS rates, since having one CS increases the likelihood of repeat CS in subsequent pregnancies. [7] However, the positive impact of increasing the rate of vaginal birth among first-time mothers on the necessity for CS may be attenuated by subsequent decreases in the rate of vaginal birth after CS (VBAC). The relationship between CS rates within relevant subpopulations and the overall CS rate at a population level is, therefore, complicated and requires longitudinal analysis that takes account of the relationship between each of these parameters over time.

The aim of this study was to estimate the relative effect of decreasing nulliparous CS rates and increasing VBAC rates on total costs of hospital births in Ireland and England/Wales, to help inform policymaking about how best to target and scale initiatives designed to reduce CS rates in these two regions.

## Methods

Decision analysis modelling was used to estimate the net present value of changes in resource use associated with decreasing CS rates among the current cohort of 18-year-old women in Ireland and England/Wales over the course of their most active childbearing years between the ages of 18 and 44 years. The analysis was carried out from the perspective of the public health system, and included the cost of all medical and surgical procedures carried out during the hospital birth episode of care, as well as hospital accommodation, as captured by diagnosis-related group (DRG) costings for each health system. [8,9] DRGs are classifications used to group together episodes of care based on the clinical profile of patients and the treatment they receive, to provide a basis for calculating costs of resource use associated with different diagnoses and procedures. They reflect the costs of treatment and accommodation for the average case within each group. Incremental costs are presented in the currency of each country (Euro for Ireland and Pounds Sterling for England/Wales) and therefore reflect the opportunity costs of maintaining current CS rates within each region rather than being directly comparable across both health systems. As no measure of the variability of DRG costs was available, these costs were varied by 20% in a probabilistic sensitivity analysis examining the impact of uncertainty about these cost estimates.

We assumed that any reduction in CS rates would be substituted with relatively low cost, uncomplicated CS births (lowest level of complexity in DRG classification). To calculate the cost of increases in the rate of vaginal birth as a consequence of any reduction in CS rates, we used the weighted average of the costs of non-CS births of all levels of complexity (spontaneous vaginal and instrumental). This is a conservative approach that minimises the cost differential by assuming that any initiative to reduce CS rates will primarily affect uncomplicated, low-cost CS births, but that the resultant additional vaginal births may involve any level of complexity or cost. Discounting (at a rate of 4%) was used to estimate the net present value of costs incurred at different time points over the 27-year time horizon modelled in the analysis, in line with Irish guidelines for health economic evaluation. [10]

Costs of antenatal and postnatal care before and after the birth episode were not included, nor did we include non-health service costs falling on women or families, such as travel or out-of-pocket costs associated with maternal morbidity, or productivity losses due to time off work. Omission of these costs represents a conservative approach that is likely to underestimate the overall difference in costs between CS and vaginal births, since CS is generally associated with greater morbidity for both mothers and babies. [5] However, because we assume that changes in mode of birth can be achieved without impacting on the provision of medically indicated CS, it is difficult to quantify how maternal and neonatal health outcomes would change within the specific cohort of women whose type of birth would be altered.

Rates of CS among primiparous women in Ireland and England/Wales were obtained from the European perinatal health report. [7] Data on CS rates among multiparous women with and without a history of CS in previous births were available for England and Wales only. In the absence of Irish data, estimates from Northern Ireland were used.

The number and timing of births in the cohort were estimated using census data from Ireland and England/Wales on the number of children born to women in each region by the age of 45 years, and mean maternal age by birth order. [11–15] Our analysis was limited to a maximum of four births per woman, and the cohort was adjusted for both the rate of multiple pregnancies and the cumulative mortality rate between 18 and 44 years in each region (0.019, 0.016 and 0.0021, 0.0029 in Ireland and England/Wales, respectively). [13,16–20] The percentage of births to women outside of this age range was 0.8% and 1.1% in Ireland and England/Wales, respectively, and the percentage of births to women who had four or more previous livebirths was 3.0% and 3.5% in Ireland and England/Wales, respectively. Adjustment for multiple pregnancies was necessary to avoid overestimating the number of hospital episodes by treating maternities with multiple births as separate birth events. Censoring at four births is a conservative measure designed to reduce the risk of overestimating the number of CS births due to an inability to model the potential impact of high parity (≥5) on mode of birth. Even when limiting to the first four births, available data does not support an assumption that mode of birth does not affect the probability of having further children. If this was the case we would expect aggregate CS rates to be positively correlated with parity, given nationally reported VBAC rates and rates of CS among women with no previous caesarean. [7] For example, the nulliparous CS rate for Ireland is 33.2%, the CS rate among those with a history of CS is 78.4% (1-VBAC rate) and the CS rate among those with no previous CS is 19.6%. Using these figures to calculate the expected CS rate among women having their second baby produces an estimate of 39.1%. However, the observed CS rate in this population is significantly lower (32.1% [8]), most likely due to differences in the average clinical or demographic profile of women having CS and vaginal births that make those having a CS less likely to have more children. Therefore to avoid overestimating the absolute number of CS we calibrated the model using observed CS rates by parity in Ireland and England/Wales. [3,8]

**Fig 1:**
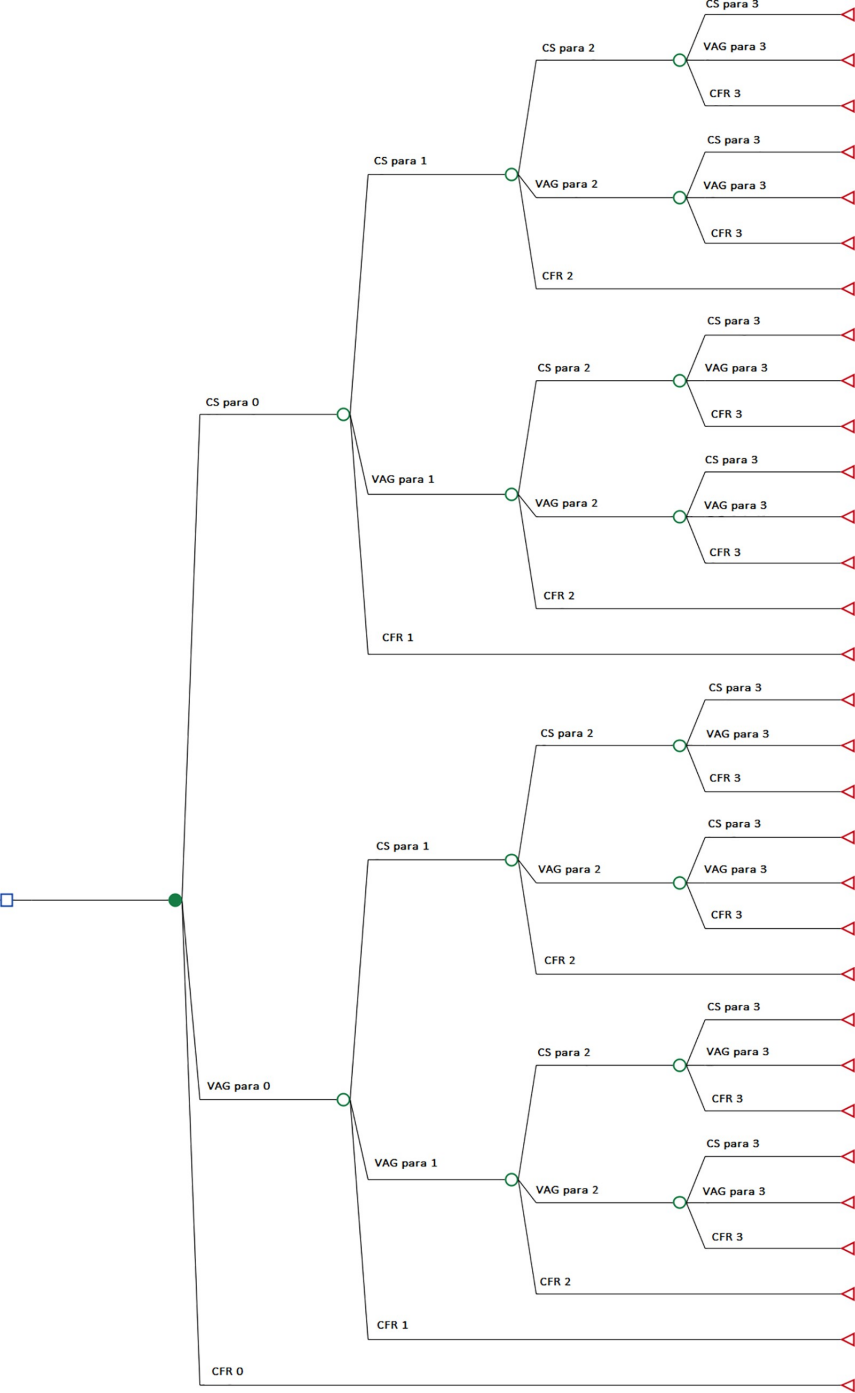
Decision tree model structure. CS caesarean section; VAG vaginal birth; CFR completed fertility rate; para parity.

The model structure is shown in Fig 1. This was used to carry out two-way deterministic sensitivity analysis estimating the cost implications of absolute improvements in primiparous CS rates and vaginal-birth-after-caesarean (VBAC) rates of 2.5%, 5%, 7.5% and 10% in both regions. To estimate the upper bound of the potential economic impact of effective policy responses, microsimulation was used to compare the current standard of care in Ireland and England/Wales with a comparator in which CS rates equalled those of the best-performing countries reported in the EuroPeristat report (Icelandic nulliparous CS rate of 18.3% and Finnish VBAC rate of 55.4%). [7] This is a more conservative estimate of what is realistically achievable in the short to medium term than using optimal CS thresholds of 10% to 15%, and may therefore provide a truer reflection of opportunity costs. In order to isolate the relative impact of nulliparous CS and VBAC rates, the rate of CS among multiparous women with no history of CS was fixed at its current level in all simulations. [7] Stochastic modelling was used to characterise the level of uncertainty associated with costs and completed fertility rate in each comparator, by sampling input parameters from their given distribution in each replication of the model. All analyses were carried out in TreeAge Pro and Stata 14. [21,22]

This study provides an analytical framework for estimating the incremental costs associated with maintaining high CS rates at a national level. Ireland and England/Wales were chosen for inclusion due to the availability of all national-level data necessary for populating the economic model, including recent DRG cost estimates and detailed census data describing the number, timing and mode of all births within the cohort of women who have reached the end of their peak childbearing years. Our model is available as a supplementary file for those interested in conducting this analysis for other health systems, using country-specific parameters (see S1 Data and S2 Data).

## Results

Analysis of primary data yielded the model parameter estimates shown in Table 1. Validation tests showing that the output of current standard of care models are consistent with observed data on the number of births in each cohort, overall national CS rates, and CS rates by parity are provided as supplementary material, along with kernel density plots showing the distribution of the sampled parameters (see S3 Data).

**Table 1:** Model parameters.

Parameter	Ireland	England & Wales	Distribution	Source
Probability of having a caesarean section for 1st baby	0.33	0.28	N/A*	Euro- Peristat 2018 [7]
Probability of having a CS after previous vaginal birth	0.20**	0.24	N/A*	
Probability of having a vaginal birth after previous CS (VBAC)	0.22**	0.27	N/A*	
Cost of uncomplicated CS	€3,272	£3,200	Gamma	HSE/NHS [8,9]
Cost of vaginal birth	€2,489	£2,478	Gamma	
Probability of having 0 children	0.18	0.18	Beta	CSO/ONS [12,15]
Probability of having 1 child	0.13	0.18	Beta	
Probability of having 2 children	0.31	0.37	Beta	
Probability of having 3 children	0.23	0.17	Beta	
Probability of having 4 or more children	0.14	0.10	Beta	
Mean (SD) age when having baby 1	30.4 (5.5)	28.8 (5.9)	Normal	CSO/ONS [11,13,14,16]
Mean (SD) age when having baby 2	32.6 (4.9)	31 (5.5)	Normal	
Mean (SD) age when having baby 3	34.0 (4.5)	32 (5.2)	Normal	
Mean (SD) age when having baby 4	34.5 (4.6)	33 (5.0)	Normal	
Cohort size***	29,204	325,315	N/A	CSO/ONS [13,16–20]

* Parameter uncertainty examined using deterministic sensitivity analysis rather than Monte Carlo simulation

** Data from Northern Ireland

*** adjusted for multiple pregnancy and cumulative mortality rate, N/A Not applicable, SD Standard Deviation; CS caesarean section, HSE Health Service Executive, NHS National Health Service, CSO Central Statistics Office (Ireland), ONS Office of National Statistics (UK)

The effect of reducing CS rates on maternity hospital costs is presented in Table 2 (for Ireland) and Table 3 (for England and Wales). This shows that relatively modest changes in nulliparous CS and VBAC rates are associated with significant reductions in hospital costs. For example, the net present value of the savings associated with a five-percentage-point change in nulliparous CS rates and VBAC rates is €1.1million and £9.8million for each successive annual cohort of 18-year-olds in Ireland and England/Wales, respectively. As expected, the relative impact of decreases in nulliparous CS rates is significantly greater than comparable reductions in VBAC rates.

**Table 2:** Impact of reducing CS rates on hospital costs and number of CSs in Ireland.

Change in costs (Euro)		Absolute change in VBAC rate				
		0.00%	2.50%	5.00%	7.50%	10.00%
Absolute change in nulliparous CS rate	0.00%	0	-104,958	-209,916	-314,875	-419,833
	-2.50%	-444,239	-542,719	-641,198	-739,677	-838,157
	-5.00%	-888,478	-980,479	-1,072,480	-1,164,480	-1,256,481
	-7.50%	-1,332,717	-1,418,239	-1,503,761	-1,589,283	-1,674,805
	-10.00%	-1,776,956	-1,856,000	-1,935,043	-2,014,086	-2,093,129

VBAC vaginal birth after caesarean, CS caesarean section

**Table 3:** Impact of reducing CS rates on hospital costs in England & Wales.

Change in costs (Pound sterling)		Absolute change in VBAC rate				
		0.00%	2.50%	5.00%	7.50%	10.00%
Absolute change in nulliparous CS rate	0.00%	0	-788,306	-1,576,612	-2,364,918	-3,153,224
	-2.50%	-4,244,922	-4,977,282	-5,709,642	-6,442,002	-7,174,363
	-5.00%	-8,489,844	-9,166,258	-9,842,672	-10,519,087	-11,195,501
	-7.50%	-12,734,765	-13,355,234	-13,975,702	-14,596,171	-15,216,639
	-10.00%	-16,979,687	-17,544,210	-18,108,732	-18,673,255	-19,237,777

VBAC vaginal birth after caesarean, CS caesarean section

To estimate the upper bounds of what could realistically be achieved, the model was run using the lowest nullipara CS rates (Iceland, 18.3%) and highest VBAC rates (Finland, 55.4%) among all countries included in the 2018 EuroPeristat report, keeping the rate of caesarean section after previous vaginal birth constant. [7] In this best-case scenario the estimated net present value of the cost savings were approximately €3.5m in Ireland, and £23.0m in England/Wales, for each annual cohort of 18 year old females (Table 4).

**Table 4:** Changes in costs and overall CS rates by achieving European-best levels of primiparous-CS and VBAC rates.

	Ireland	England/Wales
**Change in CS rate**^**1**^	-13 percentage points	-9 percentage points
**Change in costs**^**2**^	-€3,538,152	-£23,030,672

CS caesarean section, ^1^ change in overall CS rate associated with specified changes in primipara CS rates and VBAC rates combined, ^2^ Net present value of cost savings among annual cohort of 18-year-old females

**Fig 2:**
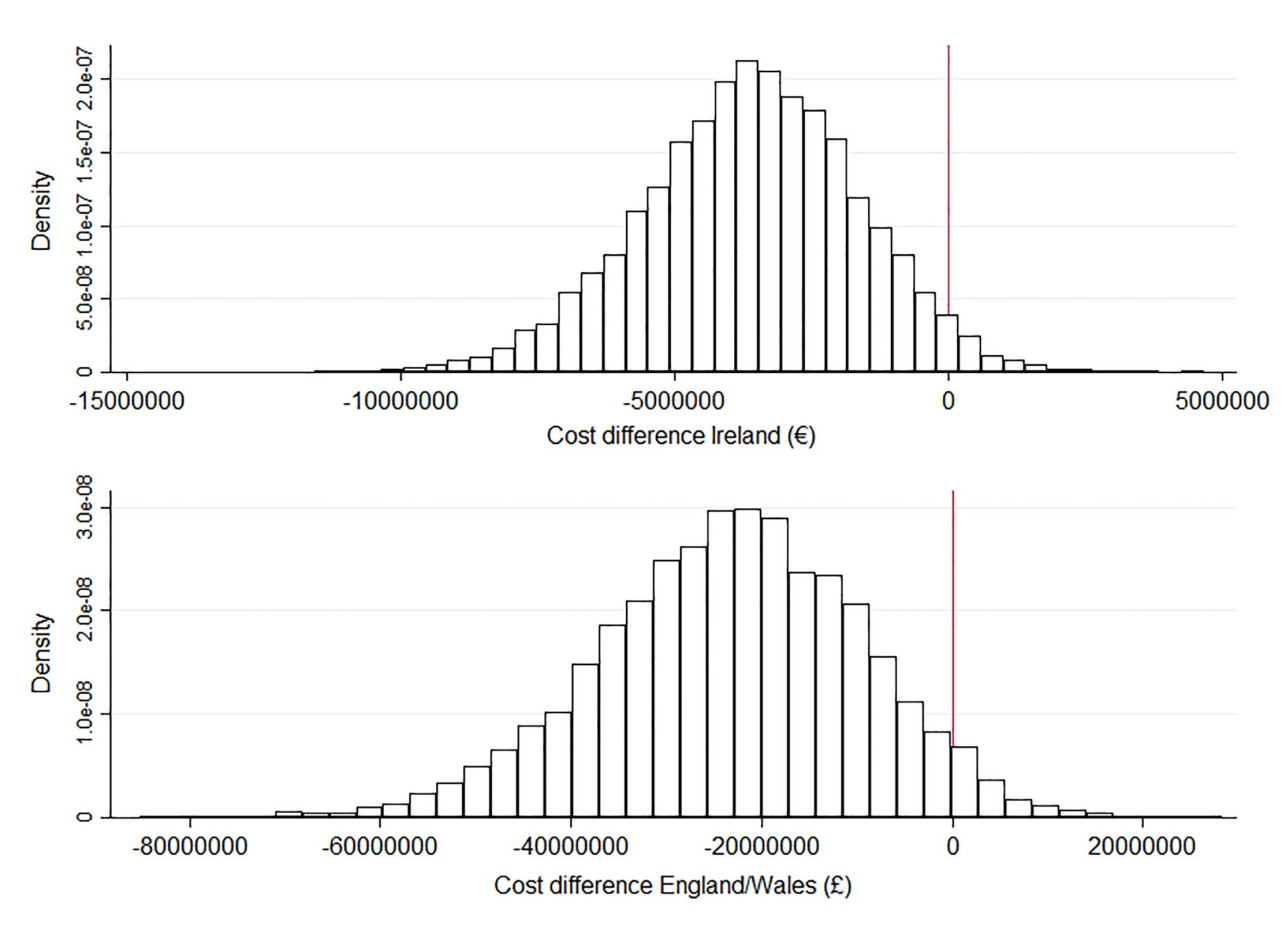
Probabilistic sensitivity analysis of changes in costs by achieving European-best levels of primiparous-CS and VBAC rates. CS caesarean section, VBAC vaginal birth after caesarean.

Fig 2 shows the results of a probabilistic sensitivity analysis examining the level of uncertainty around the mean change in costs shown in Table 4. This reflects the range of possible estimates of the net present value due to uncertainty about the cost of CS and vaginal birth and completed fertility rate. Although we incorporated a high degree of variability in DRG costs (±20%), the cost differential was positive in only 2.8% of replications of the Ireland model, and 4.2% of replications of the England/Wales model, indicating that the results are insensitive to plausible changes in unit costs in both regions.

## Discussion

Our results show that relatively small improvements in nulliparous CS and VBAC rates are associated with substantial reductions in hospital costs both in Ireland, and in England and Wales combined. Reductions in nulliparous CS rates are associated with significantly greater decreases in overall costs than comparable increases in VBAC rates. If both regions were to achieve nulliparous CS and VBAC rates comparable to those of the best-performing countries in Europe currently, the net present value of the incremental cost savings within each annual cohort of women is estimated to be €3.5m and £23.0m in Ireland and England/Wales, respectively.

These estimates are based on a longitudinal analysis of the costs within an annual cohort of women between the ages of 18 and 44, discounted to take account of when those costs are incurred over that 27-year timeframe. This approach allows for the positive effects of reductions in CS rates among first-time mothers on repeat CS rates in subsequent pregnancies to be captured, thus providing a truer reflection of the economic implications of changes in these parameters. Interpreting net present values can be challenging, so to express these results in a more intuitive way we can say that, all other things being equal, by the time these changes in primiparous CS and VBAC rates had fully filtered down to all women giving birth in a given year, undiscounted savings from the associated reduction in overall CS rates would be €6.6M and £39.6M per year in Ireland and England/Wales, respectively, compared to the current standard of care.

These findings suggest that the opportunity cost of existing rates of CS is considerable, as reflected in the significant amount of additional resources consumed for what previous analyses of optimal CS rates have found to be limited expected benefits. [4] The scale of the costs associated with maintaining current CS rates implies that even relatively costly interventions designed to reduce nulliparous CS rates or increase VBAC rates are likely to represent good value for money, as long as the net present value of their implementation was less than that of the cost saving accruing from the level of reduction they could bring about (Tables 2 and 3).

A previous analysis of worldwide CS rates estimated that the annual cost of unnecessary CS in Ireland and the UK was approximately US$14.9m and US$38.8m, respectively, in 2008. [23] This analysis was based on a cross-sectional analysis of total CS rates in each country at that time (26.2% in Ireland and 22.0% in the UK) and the excess cost was calculated as the sum of all CS procedures above a threshold of 15%, so direct comparison with our results is not possible. However, while this approach is relatively straightforward from a computational perspective, interpretation of the results is constrained by a lack of data on the relative contribution of CS rates within different subpopulations that combine to produce the overall rate, and any temporal effect these may have into the future that could affect the feasibility of reaching the 15% threshold in each country. It is also important to note that the 10% to 15% threshold is based on an analysis of mortality outcomes only, as insufficient data was available to examine the relationship between population CS rates and maternal and child morbidity, or other social or psychological outcomes related to mode of birth. [4] By basing the best-case scenario on currently observed nulliparous CS and VBAC rates in Europe, we offer a more pragmatic estimate of the scale of savings that could potentially be achieved.

As far as we are aware, ours is the first longitudinal analysis of the economic implications of plausible changes in the nulliparous CS and VBAC rates in multiple health systems. As such, it contributes new findings to inform priority setting within the health policy agenda, as well as generating valuable new information about how best to target and scale initiatives designed to stem the sustained increased in CS rates.

### Limitations

There are several important limitations that must be considered when interpreting the results. Only the cost of the hospital birth episode is included in the analysis. We do not estimate any subsequent changes in clinical outcomes among mothers and children as a result of changes in mode of birth, so any additional costs associated with treating these are excluded. Any costs that fall on women, children and families, such as productivity losses, costs of informal care, or out-of-pocket payments for services not provided by the public health system, were also omitted. Given the likelihood that CS is associated with higher postpartum morbidity than vaginal birth, this probably means our results are an underestimate, provided any reduction in CS can be achieved without any decrease in the number of medically indicated CS births. [5] However, even without reducing access to medically indicated CS, decreasing the overall rate of CS may lead to increased morbidity and associated cost increases for at least some types of health problems, such as pelvic floor function problems. [24]

Incremental cost estimates require data on the total population of women in each region, as well as the expected number and order of children that will be born to women within that cohort. We used census data on the total number of women aged 18 years in Ireland and England/Wales in 2016, and adjusted for the background mortality rate and rate of multiple pregnancies to avoid overestimating the number of hospital birth episodes. However, we did not include stillbirths in the analysis, nor did we include births of fifth or subsequent children. Rates of stillbirth in Ireland and England/Wales in 2016 were 2.7 and 4.4 per 1,000, respectively, and the percentage of births to mothers with 4 previous children was 3.0% in Ireland and 3.5% in England/Wales in that year. [3,11,25] Failure to include these in the model is unlikely to affect the results given the relatively low numbers, combined with the limited impact that any measures to change overall CS rates are likely to have within these groups. We did not assess the potential impact of future trends in CS rates, fertility rates and maternal age in successive cohorts after 2016. Instead, this analysis assumed that these parameters remain fixed within the modelled cohort. In addition, as no data was available for Ireland on CS rates in multiparous women with and without a history of CS, data for Northern Ireland were used to estimate these two parameters in the model. While the overall rate of CS among multiparous women in Northern Ireland (29.6%) is more closely aligned with Ireland (30.1%) than England/Wales (26.6%), in the absence of additional data we assumed that CS rates among multiparous women with and without a history of CS are also comparable. [7]

Estimates of the costs of each type of birth were obtained from DRG systems within each public health system. Use of DRG costs can be misleading if there are significant differences in the case mix between the current standard of care and modelled comparators. For instance, if the clinical profile of CS cases averted in comparators with lower CS rates was somehow different from the clinical profile of CS cases in standard care then this could bias our estimates. To mitigate this, we took the conservative approach of assuming that only CS cases of the lowest complexity (and therefore costs) would be substituted, and that the additional vaginal births could be of any level of complexity. It must also be noted that there are differences between the DRG systems in use in Ireland and England/Wales that preclude direct comparisons of the costs across regions. Ireland uses the Australian Refined Diagnosis Related Group (AR-DRG) system, whereas England/Wales use Healthcare Resource Groups (HRGs). [26,27] While both these systems seek to group clinically similar treatments which consume comparable healthcare resources, there are differences in how births are classified in each and the least complex CS birth category is not necessarily the same in both. The discount rate of 4% used in the analysis was taken from Irish national guidelines, which differs from the 3.5% discount rate recommended in the UK. [10,28] Given the degree of similarity between the two, use of the UK discount rate is not expected to significantly affect the results.

Costs associated with professional indemnity and medico-legal claims for harm caused by birth injury were not included. For these to be eligible, they needed to reflect an opportunity cost from the perspective of the public health system, and sufficient data needed to be available to address the question of attribution. Neither of these conditions are met in the context of estimating the incremental costs of unnecessary CS. Costs in respect of birth injury or medical negligence claims may include legal fees, special damages to compensate for costs associated with medical care and loss of earnings, general damages to compensate for non-pecuniary losses such as pain and suffering, and sometimes punitive or exemplary damages to punish poor professional practice or deter future negligence. Of these, only damages to cover the medical treatment represent an opportunity cost from the perspective of the public health service, rather than direct non-healthcare costs that would be relevant when taking a societal, rather than a health service, perspective. Even if it was somehow possible to disentangle these costs it is unclear how they should be attributed. Others have attempted to apportion overall indemnity and litigation costs by mode of birth and add a premium to each case to reflect the average cost per birth. [29] We find this unsatisfactory due to the difficulty in accurately allocating these costs based on limited information about the clinical circumstances of birth injury cases, as well as concerns about the assumption that these costs can be distributed evenly to all cases, which implies that changes in litigation and birth injury claims costs will respond linearly to changes in national CS and vaginal birth rates. The relationship between mode of birth at a population level and total costs of claims is unlikely to be that simple. While there is some evidence to show that higher CS rates are associated with lower rates of malpractice suits in the US, we were unable to identify any empirical analysis to support the idea that increasing CS rates drive down overall medicolegal claims costs within health services. [30] In both Ireland and the UK, claims related to cerebral palsy are by far the biggest driver of costs. [31,32] However, a systematic review of the association between CS and cerebral palsy found no evidence that CS reduces the risk of this outcome. [33]

Finally, this analysis is exploratory in nature and does not attempt to estimate the costs or clinical effectiveness of interventions designed to reduce CS rates. Rather, it is designed to indicate the cost implications of relatively modest decreases in CS rates among first-time mothers or those with a history of CS, as well as more transformative changes that bring Ireland and England/Wales in line with the best-performing countries in Europe. Experience has shown that the task of implementing health policy that manages to realise these goals is challenging and may require significant resources. [34] Our analysis provides a conservative estimate of the savings to the public health system associated with reducing CS rate if these reductions could be achieved at zero cost. This provides valuable information to policymakers faced with a range of available alternatives with different resource requirements and expected effect sizes.

## Conclusion

The economic benefits of increasing rates of vaginal birth are considerable, and even relatively modest decreases in the rate of CS among first-time mothers and those with a history of CS are associated with significant reductions in hospital delivery costs in Ireland and England/Wales. Reductions in CS rates among first-time mothers are associated with a greater payoff than comparable increases in VBAC rates.

## Supporting information

S1 Data (TREX)

S2 Data (TREX)

S3 Data (PDF)
