# Supplementary material for: Economic implications of reducing caesarean section rates – Analysis of two health systems
Source: PLoS One. 2020 Jul 28;15(7):e0228309. doi: 10.1371/journal.pone.0228309 (PMC7386590; doi:10.1371/journal.pone.0228309)
Supplement: S3 Data — (PDF) [file pone.0228309.s003.pdf]

Supplementary material

Model Validation

i. Proportion of women with completed fertility rate (CFR) of 0 to ≥4

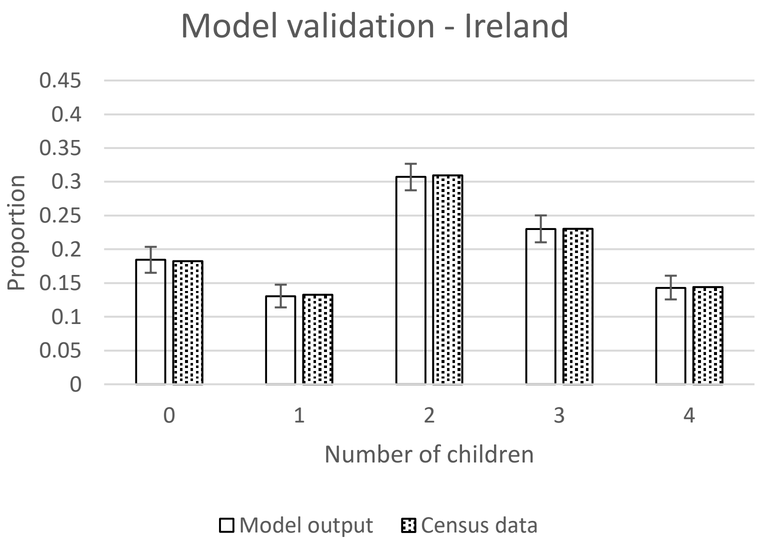

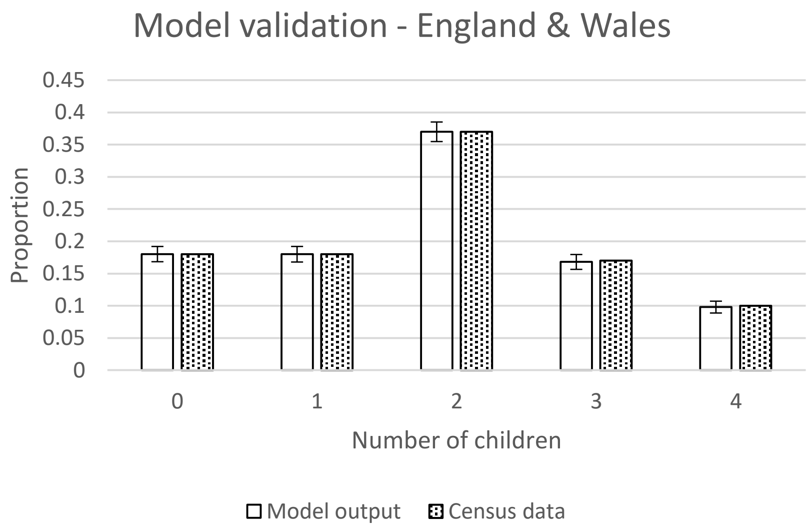

ii. Comparison of modelled average caesarean section rates (overall and by parity), versus observed data(1, 2)

	**Overall CS rate**	**First baby**	**Second baby**	**Third baby**	**Fourth baby**
**Ireland (Observed)**	0.313	0.332	0.321	0.311	0.284
**Ireland (Modelled)**	0.321	0.332	0.323	0.310	0.279
**England/Wales (Observed)**	0.270	0.281	0.273	0.274	0.270
**England/Wales (Modelled)**	0.277	0.281	0.271	0.272	0.272

CS caesarean section

iii. Kernel density plots of sampled model inputs

Ireland model

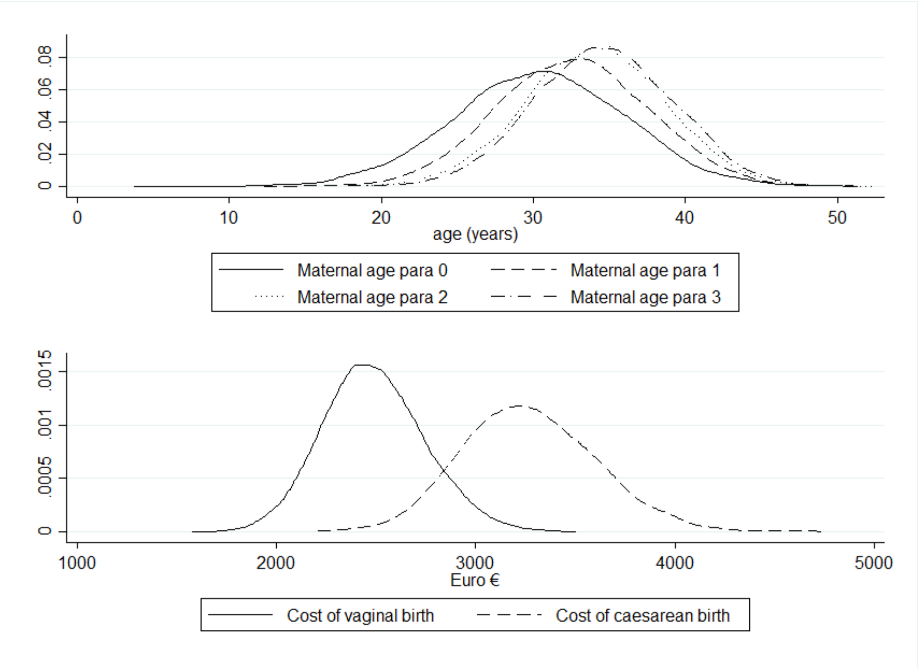

England/Wales model

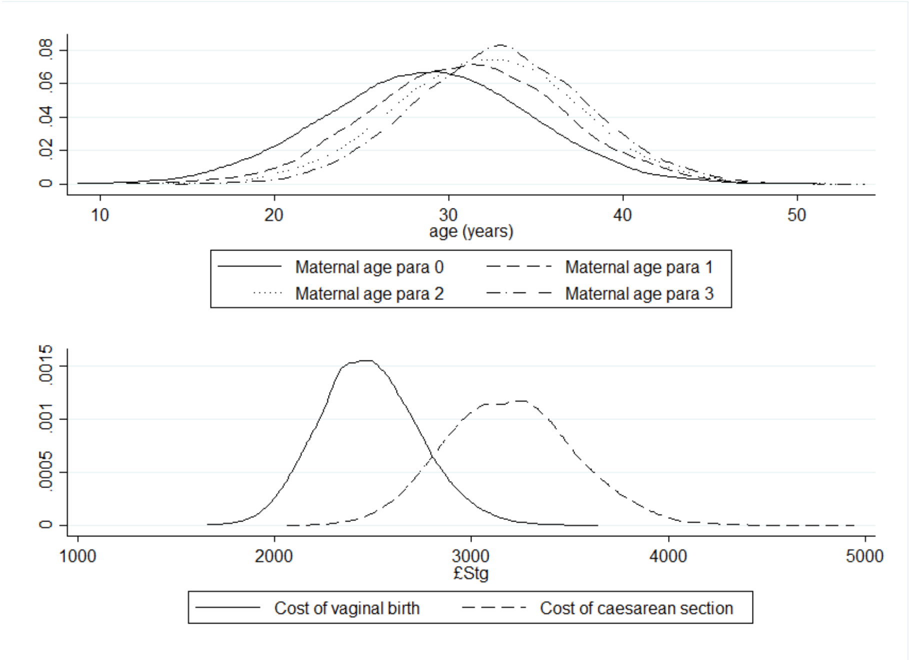

1. Healthcare Pricing Office. HIPE Reporting Database. Dublin, Ireland Health Service Executive; 2017.
2. NHS Digital. NHS Maternity Statistics 2016-2017. England: National Health Service; 2017.
